# HER2 Basolateral versus Circumferential IHC Expression Is Dependent on Polarity and Differentiation of Epithelial Cells in Gastric/GE Adenocarcinoma

**DOI:** 10.1155/2018/6246493

**Published:** 2018-07-24

**Authors:** Shahid Pervez, Sidra Arshad, Brooj Abro

**Affiliations:** Department of Pathology & Laboratory Medicine, Aga Khan University Hospital, Stadium Road, Karachi 74800, Pakistan

## Abstract

**Aim:**

Antigenic expression in epithelial cells can be heterogeneous which may pose a problem in immunohistochemical (IHC) analysis of tumor markers, in particular, predictive markers like HER2. Studies have shown that epithelial cells have distinct apical and basolateral domains which are separated by tight junctions. The cell membrane in these two domains has a different composition of macromolecules and hence can have variable antigen expression on immunohistochemistry. In our study, we aimed to investigate this phenomenon of basolateral versus circumferential IHC staining of HER2 in gastric/GE adenocarcinoma.

**Methods:**

We selected 45 cases of gastric/GE adenocarcinoma and evaluated equal number of specimens (15 each) showing well-differentiated, moderately differentiated, and poorly differentiated morphology. All cases had 3+ HER2 score as per CAP guidelines. HER2-membrane staining pattern in all specimens was analyzed.

**Results:**

Cases with well-differentiated morphology showed only basolateral or lateral membrane staining in most cases. Poorly differentiated adenocarcinoma samples showed circumferential staining (both basolateral and luminal) in all cases with highly significant p value. Mixed staining pattern was observed in moderately differentiated cases. Diffuse expression of E-cadherin in well-differentiated adenocarcinoma and loss in poorly differentiated tumors were also statistically significant.

**Conclusion:**

These findings suggest that HER2 in gastric epithelium has a polarized distribution which is maintained by the fence function of tight junctions. With progression to high grade cancer, the glandular structural differentiation in gastric mucosa is lost, along with disruption of tight junctions. This leads to loss of cell polarity and migration of antigens across the membrane.

## 1. Introduction

In epithelial tissue, cells characteristically have cell to cell adhesion and an apicobasal membrane polarity; i.e., the apical and basal membranes have a distinct set of macromolecules. It has been postulated that tight junctions are important for maintaining cell polarity [1]. In epithelial tissue, tight junctions are intercellular junctional complexes along the lateral border of cells forming tight adhesion between cells and maintaining tissue integrity [2]. Tight junctions perform various functions; two important ones are (i) barrier function, i.e., allowing selective molecules to diffuse through intercellular space, and (ii) fence function, i.e., dividing the cell membrane into apical and basolateral surfaces, providing and maintaining a polarized distribution of certain membrane components across the cell [3, 4].

Tight junctions can become dysfunctional in cancer and this plays an important role in tumorigenesis, tumor progression, and metastasis [5]. Loss of tight junctions can result in disruption of the apicobasal polarity in epithelial cells and lead to a change in antigen expression by the cell membrane [6]. In cancer diagnostics, it is common to use immunohistochemical (IHC) techniques to stain various membrane antigens specific to a particular type of tumor. Any changes in the polarity of cell membrane can cause changes in the staining pattern.

This study was conducted to study the variable staining pattern of HER2 (human epidermal growth factor receptor 2) in gastric/gastroesophageal (GE) adenocarcinoma. Level of E-cadherin expression in tumors of various grades was also taken into account. HER2 is a plasma membrane bound receptor and is known to be amplified in certain tumors. Breast cancer is most well-known for this, about 15 to 30% are positive for HER2 overexpression, and this indicates a worse prognosis when compared to HER2 negative breast cancers [7, 8]. The prognosis of these patients can be improved by adding trastuzumab to the treatment regimen. In recent years, HER2 has also been shown to play an important role in gastric and gastroesophageal adenocarcinoma and in breast cancer and some studies have shown that this indicates a poor prognosis [9, 10] which again can be improved by using trastuzumab. On an average about 15-18% of gastric and GE cancer cases are HER2 positive [11]. The ToGA trial in 2010 demonstrated an improved survival rate in patients with HER2-positive advanced gastric and GE tumors, treated with trastuzumab, an anti-HER2 monoclonal antibody [12]. Hence, HER2 testing in such cases is imperative to select patients for this beneficial targeted therapy. In gastric cancer, the membranous distribution of the HER2 molecule is mostly basolateral, in contrast to circumferential membrane staining HER2 3+ breast cancer [13]. In this study we have used HER2 IHC staining for different histologic grades of gastric adenocarcinoma to investigate differences in patterns of expression of HER2.

## 2. Methods

### 2.1. Tissue Specimens

We selected cases for this study from the Pathology Department of Aga Khan University Hospital, Karachi, Pakistan. Surgical resection specimens from patients diagnosed with intestinal type gastric and GE adenocarcinoma, showing 3 + HER2 IHC staining, were selected. The IHC semiquantitative scoring on the specimens was done as per College of American Pathologists (CAP) guidelines (Rüschoff, Josef et al. 2010). This is universally accepted protocol for scoring of HER2 receptors in gastric and breast biopsies. Strong staining observed at low power in greater than or equal to 10% of tumor cells was considered to have 3 + HER2 score. The histologic classification of the specimens selected was based on Lauren's criteria of gastric adenocarcinoma. The specimens included in this study were consistent with intestinal type morphology as mentioned in Lauren's classification [14]. Only very well-differentiated cases with well-preserved polarity of neoplastic cells were kept and classified as well-differentiated cases, while cases with signet ring morphology were excluded.

### 2.2. Immunohistochemistry

HER2-protein expression in tumor specimens was analyzed using Hercept test according to manufacturer's recommended protocol (DAKO, Carpentaria, CA). These samples were fixed in 10% buffered formalin, for not more than 72 hours. Sections of resected specimens were cut with 3-5 um thickness and transferred to antigen retrieval solution (Tris-EDTA Ph 9). Following this they were placed in oven at 65 degrees Celsius for 30 minutes and transferred to P.T Link for 40 minutes, where temperature was increased from 85 degrees Celsius to 97 degrees Celsius and then back to 85. Specimens were than washed in buffer for 5 minutes. Rabbit polyclonal antibody (clone: A0485, DAKO) was applied at a dilution of 1:1000 for 20 minutes and then detected by Envision System (DAKO). Positive and negative controls provided in the kit were added to each batch of samples. Ten cases from both well-differentiated and poorly differentiated adenocarcinomas were also stained with a monoclonal antibody against anti-human E-cadherin (clone NCH-38, DAKO) to assess its expression as E-cadherin is a transmembrane cell adhesion molecule involved in cell adhesion of epithelial cells and establishment of epithelial polarization.

The HER2 score was established as per CAP guidelines; membrane staining (basolateral, lateral, or circumferential) visible at low magnification (10x) in equal to or greater than 10% of tumor cells in surgical specimens was considered 3+.

## 3. Results

**Figure 1:**
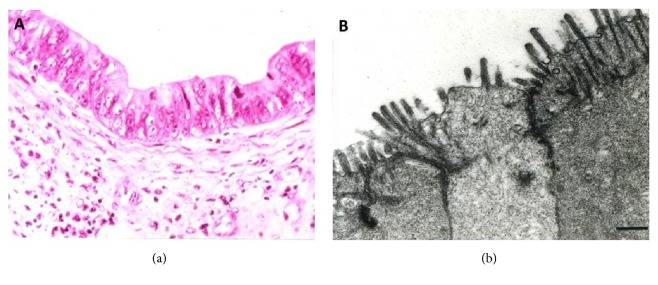
(a) Well-differentiated gastric adenocarcinoma on H&E stain. (b) EM showing functional tight junctions in the specimen.

**Figure 2:**
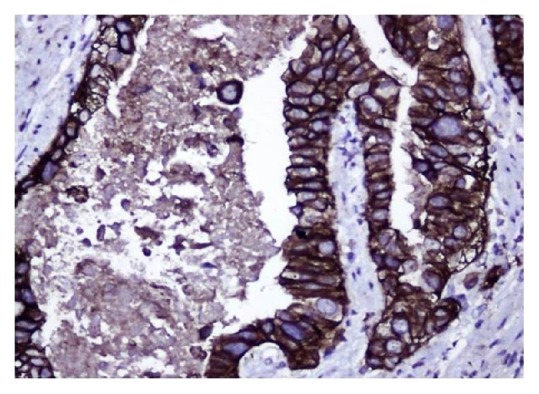
HER2 immunohistochemical staining of well-differentiated adenocarcinoma (note only basolateral staining and no luminal staining).

**Figure 3:**
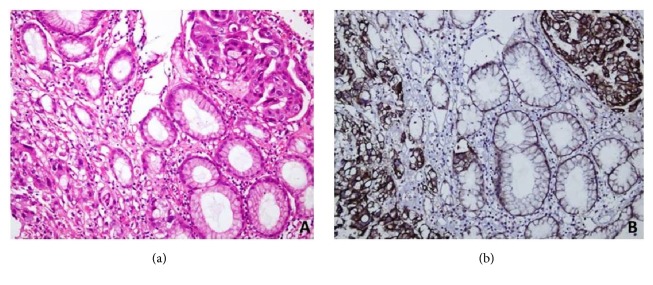
(a) H&E of poorly differentiated gastric adenocarcinoma. (b) HER2 immunohistochemical staining showing circumferential staining of tumor cells.

We observed that well-differentiated gastric and GE adenocarcinoma with prominent glandular differentiation (Figure 1) largely expressed only basolateral HER2 IHC staining with no luminal staining (Figure 2). Exclusive basolateral and lateral staining was seen in 10 out of 15 specimens (66.6%), with no circumferential staining. Meanwhile the remaining 05 cases showed circumferential staining in less than 10% of tumor cells. In contrast to this, poorly differentiated adenocarcinomas expressed circumferential staining, both basolateral and luminal (Figure 3), consistently in all 15 specimens.

**Figure 4:**
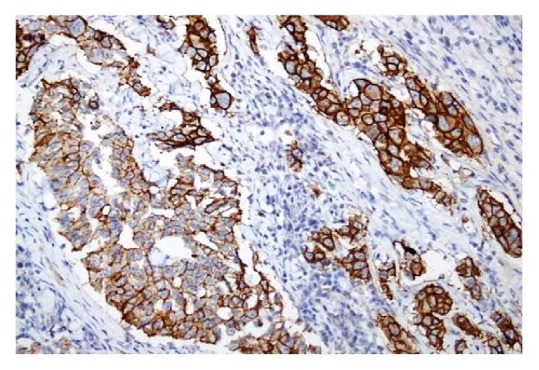
HER2 immunohistochemical staining of gastric adenocarcinoma showing antigen heterogeneity (note 2+ and 3+ membrane staining in the same tumor).

Statistical P value was highly significant (P<0.001) when staining pattern of well-differentiated group was compared to poorly differentiated group. Moderately differentiated ones showed a mixed staining pattern. These showed predominantly basolateral staining; however circumferential staining was also seen in detached clusters of tumor cells.  Figure 4 shows heterogenous pattern of circumferential membrane staining in poorly differentiated gastric adenocarcinoma (both 2+ and 3+ HER2 score in cells from the same tumor).

**Figure 5:**
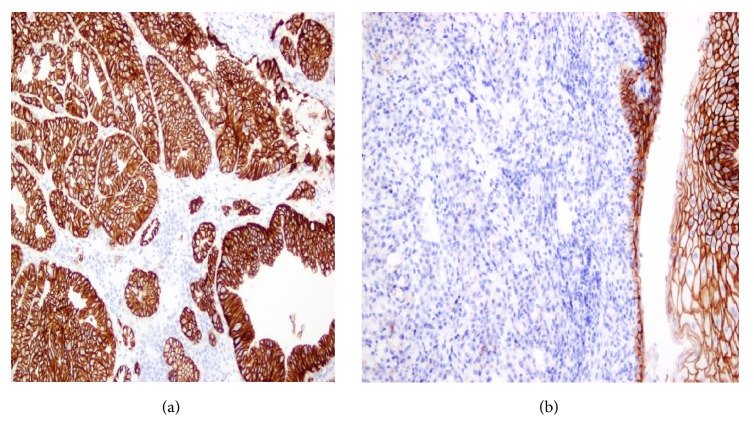
(a) Well-differentiated adenocarcinoma of stomach stained with E-cadherin showing diffuse strong membrane staining. (b) Poorly differentiated adenocarcinoma of GE junction showing complete loss of E-cadherin (please note diffuse strong membrane staining of overlying normal appearing squamous epithelium).

E-cadherin expression was diffuse and strong in all 10/10 cases of well-differentiated adenocarcinoma, while 8/10 poorly differentiated cases showed complete or near complete loss of E-cadherin. This correlation was also statistically significant with a p vale of <0.001.  Figure 5(a) shows a well-differentiated adenocarcinoma of stomach stained with E-cadherin showing diffuse strong membrane staining. In contrast Figure 5(b) shows a poorly differentiated adenocarcinoma of GE junction showing complete loss of E-cadherin (please note diffuse strong membrane staining of overlying normal appearing squamous epithelium).

## 4. Discussion

Tight junctions are a defining characteristic of polarized epithelial cells (Figure 1), forming functionally distinct apical and basolateral domains [15–17]. These two domains have different functions and hence their membrane surfaces differ in composition [18]. Tight junctions prevent the intermixing of molecules between the apical and basolateral surfaces of an epithelial cell. Multiple abnormalities have been elucidated in junctional complexes of cancerous tissues [19, 20]. Various scientific studies have shown that loss of tight junction components can lead to a more homogenous spread of antigens across the cell membrane [21–23].

Immunohistochemical techniques are widely used against epithelial antigens to aid in cancer diagnosis [24]; however, tumor antigenic heterogeneity can cause uncertainties [25]. Many studies have identified heterogeneous expression of antigens in normal epithelium and cancer tissues by using monoclonal antibodies [26–28]. Tumor differentiation and spatial configuration of cells in a tissue sample can cause this variable expression of antigens [26, 29]. In gastric adenocarcinoma, HER2 expression can be quite heterogeneous and mostly there is incomplete membrane staining, which is the basis for a different criterion for HER2 scoring in gastric cancer when compared to breast cancer [11]. Similarly, many studies have shown the variable loss of E-cadherin expression in gastric adenocarcinomas; as the tumor grade increases and becomes poorly differentiated, the expression is lost. Most of the diffuse type (signet ring cell) tumors show the highest degree of E-cadherin loss [30–32].

In this study, we have identified a variable pattern of HER2 and E-cadherin expression at various grades of histological differentiation in gastric and GE adenocarcinoma. Our study shows that HER2 has a polarized distribution in gastric/GE neoplastic epithelium which becomes disrupted and more homogenous in poorly differentiated cancer due to loss of tight junctions. Tight junctions have several membrane and cytoplasmic proteins [33]; some of these such as occludins, claudins, and zona occludins (ZO proteins) have been well studied [34–38]. Aberrant expression of claudins has been noted in epithelial cancer tissues, with both up- and downregulation of certain claudins in different types of cancers [20, 39, 40]. Kimura et al. studied the expression of occludin and ZO-1 in gastrointestinal tract (GI) epithelium in both normal and cancer tissue. They identified that these components are important for the formation and maintenance of well-formed glandular structures that are present in GI mucosa. Their investigation showed a reduced expression of occludin and ZO-1 in poorly differentiated gastric and colorectal adenocarcinoma [41].

Tobioka et al. explored the staining pattern of carcinoembryonic antigen (CEA) in colon cancer and its correlation with expression of occludin. The study showed an apical distribution of CEA in well-differentiated cancer tissue, which also had a high expression of occludin. Contrary to this, in the poorly differentiated counterparts, a circumferential staining of CEA was observed which correlated with loss of occludin expression [42]. In a previous study (Pervez et al. 1998) we observed a similar pattern with epithelial membrane antigen (EMA) antibody [26]. This antibody showed a homogenous membrane staining in poorly differentiated adenocarcinoma of ovary, breast, and colon, whereas a heterogenous pattern (predominantly apical/luminal) was observed in well-differentiated tumors.

Our current study explores the concept behind basolateral versus circumferential HER2 expression in gastric and GE cancer using IHC technique. Well-differentiated tumor specimens showed a prominent if not exclusive basolateral staining pattern. The loss of differentiation in cancer tissue correlated with a circumferential staining of HER2, indicating a loss in cell polarity leading to migration of this antigen along the cell membranes, also correlating with the loss of E-cadherin expression in poorly differentiated tumor cells.

In summary, our study shows that antigenic expression in tumor cells is not static but rather a dynamic property which can vary with tumor differentiation, cell adhesion, and tight junction integrity. To the best of our knowledge this is the first study which shows the frequency of circumferential versus basolateral HER2 IHC staining in gastric adenocarcinoma and the plausible rational behind it. Even though the conclusion in our study is based purely on our observations, multiple previous studies do support this phenomenon of heterogenous expression in various other types of antigens and have found a link between expression pattern of membrane antigens with integrity of tight junctions.

This peculiar basolateral or lateral staining along with circumferential staining pattern forms the basis of HER2 scoring for HER2 positivity and patient selection for Trastuzumab therapy nationwide. An interesting question also arises: whether variable antigen expression may also have implications on antibody localization and hence effectiveness of anti-HER2 therapy as after all accessibility and binding of injected antibody do depend on concentration as well as location of antigen on tumor cell membranes. This needs experimentation on animal models.

## 5. Conclusion

These findings suggest that HER2 in gastric epithelium has a polarized distribution which is maintained by the fence function of tight junctions and adhesion molecules like E-cadherin on epithelial cells. With progression to high grade cancer, the glandular structural differentiation in gastric mucosa is lost, along with disruption of tight junctions. This leads to loss of cell polarity and migration of antigens across the membrane.
